# Physiological effects of lung-protective ventilation in patients with lung fibrosis and usual interstitial pneumonia pattern versus primary ARDS: a matched-control study

**DOI:** 10.1186/s13054-023-04682-5

**Published:** 2023-10-18

**Authors:** Roberto Tonelli, Salvatore Grasso, Andrea Cortegiani, Lorenzo Ball, Ivana Castaniere, Luca Tabbì, Riccardo Fantini, Dario Andrisani, Filippo Gozzi, Antonio Moretti, Giulia Bruzzi, Linda Manicardi, Stefania Cerri, Anna Valeria Samarelli, Giulia Raineri, Francesco Murgolo, Andrea Carzoli, Rossella Di Mussi, Stefano Busani, Raffaella Rizzoni, Giacomo Grasselli, Enrico Clini, Alessandro Marchioni

**Affiliations:** 1 Respiratory Diseases Unit, Department of Medical and Surgical Sciences, University Hospital of Modena, University of Modena Reggio Emilia, Modena, Italy; 2 Clinical and Experimental Medicine PhD Program, University of Modena Reggio Emilia, Modena, Italy; 3 Laboratory of Cell Therapies and Respiratory Medicine, Department of Medical and Surgical Sciences for Children and Adults, University Hospital of Modena, Modena, Italy; 4 Dipartimento di Medicina di Precisione e Rigenerativa e Area Ionica (DiMePre-J) Sezione di Anestesiologia e Rianimazione, Università degli Studi di Bari “Aldo Moro”, Ospedale Policlinico, Bari, Italy; 5 Department of Surgical, Oncological and Oral Science (Di.Chir.On.S.), University of Palermo, Palermo, Italy; 6 Department of Anesthesia, Intensive Care and Emergency, Policlinico Paolo Giaccone, Palermo, Italy; 7 Department of Surgical Sciences and Integrated Diagnostics, University of Genoa, Genoa, Italy; 8 Anesthesia and Critical Care, San Martino Policlinico Hospital, IRCCS for Oncology and Neurosciences, Genoa, Italy; 9 University Hospital of Modena, Modena, Italy; 10 Department of Engineering, University of Ferrara, Via Saragat 1, Ferrara, Italy; 11 Dipartimento di Anestesia, Rianimazione e Emergenza-Urgenza, Fondazione IRCCS (Istituto di Ricovero e Cura a Carattere Scientifico) Ca’ Granda Ospedale Maggiore Policlinico, Milan, Italy; 12 Department of Pathophysiology and Transplantation, University of Milan, Milan, Italy; 13 Laboratory of Experimental Pneumology, University Hospital of Modena, Policlinico, UNIMORE, Università degli Studi di Modena e Reggio Emilia, Modena, Italy

**Keywords:** Interstitial lung disease, Pulmonary fibrosis, Usual interstitial pneumonia, Acute respiratory failure, ARDS, Lung elastance, Lung elastance, Respiratory mechanics, End-inspiratory transpulmonary pressure, End-expiratory transpulmonary pressure, Invasive mechanical ventilation, VILI, Transpulmonary pressure

## Abstract

**Background:**

Although patients with interstitial pneumonia pattern (ILD-UIP) and acute exacerbation (AE) leading to severe acute respiratory failure may require invasive mechanical ventilation (MV), physiological data on lung mechanics during MV are lacking. We aimed at describing the physiological effect of lung-protective ventilation in patients with AE-ILD-UIP compared with primary ARDS.

**Methods:**

Partitioned lung and chest wall mechanics were assessed in a series of AE-ILD-UIP patients matched 1:1 with primary ARDS as controls (based on BMI and PaO_2_/FiO_2_ ratio). Three PEEP levels (zero = *ZEEP*, 4–8 cmH_2_O = *PEEP*_LOW_, and titrated to achieve positive end-expiratory transpulmonary pressure *P*_L,EE_ = *PEEP*_TITRATED_) were used for measurements.

**Results:**

Ten AE-ILD-UIP patients and 10 matched ARDS were included. In AE-ILD-UIP median *P*_L,EE_ at ZEEP was − 4.3 [− 7.6– − 2.3] cmH_2_O and lung elastance (*E*_L_) 44 [40–51] cmH_2_O/L. At PEEP_LOW_, *P*_L,EE_ remained negative and *E*_L_ did not change (*p* = 0.995) versus ZEEP. At PEEP_TITRATED_, *P*_L,EE_ increased to 0.8 [0.3–1.5] cmH_2_O and *E*_L_ to 49 [43–59] (*p* = 0.004 and *p* < 0.001 compared to ZEEP and PEEP_LOW_, respectively). Δ*P*_L_ decreased at PEEP_LOW_ (*p* = 0.018) and increased at PEEP_TITRATED_ (*p* = 0.003). In matched ARDS control PEEP titration to obtain a positive *P*_L,EE_ did not result in significant changes in *E*_L_ and Δ*P*_L_.

**Conclusions:**

In mechanically ventilated AE-ILD-UIP patients, differently than in patients with primary ARDS, PEEP titrated to obtain a positive *P*_L,EE_ significantly worsened lung mechanics.

**Supplementary Information:**

The online version contains supplementary material available at 10.1186/s13054-023-04682-5.

## Background

Patients with interstitial lung disease and usual interstitial pneumonia pattern (ILD-UIP) may experience severe acute hypoxic respiratory failure (AHRF) during acute exacerbations (AE-ILD-UIP) [1], requiring invasive respiratory support (MV) [2]; nevertheless, the mortality following MV exceeds 80% [3]. Patho-physiologically, AE-ILD resembles acute respiratory distress syndrome (ARDS) with diffuse alveolar damage (DAD), superimposed on a background of lung fibrosis [4].

In ARDS, lung-protective MV strategies contributed to mitigate ventilatory induced lung injury (VILI), thus decreasing mortality [5, 6]. Talmor and coworkers showed that an esophageal pressure (*P*_es_)-guided positive end-expiratory pressure (PEEP) titration to obtain a positive end-expiratory transpulmonary pressure (*P*_L,EE_) is useful to recruit dependent lung regions, improve lung mechanics and minimize atelectrauma in these patients [7].

Retrospective data suggest that patients with AE-ILD are particularly susceptible to stress and strain, and hence at higher risk of VILI [8]. Thus, it seems straightforward to use lung-protective ventilatory strategies in these patients. However, little is known on *P*_L,EE_ in patients with AE-ILD-UIP and even less on the potential impact of lung-protective strategies aimed at maintaining positive *P*_L,EE_.

We studied the impact of different PEEP settings (zero PEEP [ZEEP], PEEP_LOW_ and PEEP_TITRATED_ to obtain positive *P*_L,EE_) in patients with AE-ILD-UIP, compared it with matched primary ARDS controls. We hypothesized that the impact of PEEP titration on partitioned respiratory mechanics could be different between the groups.

## Methods

### Study setting and population

The study (ClinicalTrial.gov ID NCT05098717) was carried out at the Respiratory Intensive Care Unit (RICU) of the University Hospital of Modena (Italy) in accordance with the Ethics Committee “Area Vasta Emilia Nord” approval (registered protocol number 327/2022). Informed consent to divulgate data was obtained from participants or their relatives, as appropriate.

Patients with AE-ILD-UIP developing AHRF and consecutively admitted to the RICU (August 1st, 2016, to July 1st, 2022) were eligible for enrollment. Inclusion criteria were age > 18 years; established diagnosis of ILD with a UIP pattern on a high-resolution computed tomography scan; invasive MV in volume-controlled mode. Patients suffering from chronic obstructive pulmonary disease, neuromuscular disease and chest wall deformities were excluded. AE-ILD-UIP were then matched 1:1 by body mass index, PaO_2_/FIO_2_ and acute physiology and chronic health evaluation (APACHE) II score at admission, to a group of patients with primary ARDS under MV extracted from our dataset over the same period.

### Study procedures and aim

According to our institutional protocol, patients with AE-ILD-UIP or ARDS requiring MV were submitted to a partitioned respiratory mechanics measurements within 24 h from admission during three different lung-protective strategies including low *V*_T_ (6 ml/Kg/PBW) and three consecutive PEEP levels, i.e., 0 cmH_2_O (ZEEP), 4–8 cmH_2_O (PEEP_LOW_), and *P*_es_-guided titration to obtain positive *P*_L,EE_ (PEEP_TITRATED_). At each phase, PEEP level was maintained for 30 min before recording all respiratory parameters and arterial blood sampling (see details in Additional file 1: Supplement [9, 10]).

The aim was to report measures of partitioned respiratory mechanics under lung-protective MV at different PEEP levels in patients with AE-ILD-UIP compared with ARDS.

### Data collection and analysis plan

Demographics, clinical characteristics, available pulmonary functions tests within 12 months before AE-ILD and partitioned respiratory mechanics were collected.

Data were displayed as median and interquartile range for continuous variables and numbers and percentages for dichotomous variables. Group comparison was built using a one-to-one propensity score matching procedure with the nearest-neighbor method without replacement (caliper = 0.2). Comparison between continuous variables was performed with Wilcoxon and Wilcoxon signed-rank tests. Dichotomous variables were compared using the *χ*^2^ test. Kruskal–Wallis was used to test as an interaction for whether the change in respiratory mechanics and physiological variables according to PEEP settings was different between groups. Statistics was performed using SPSS version 25.0 with PSMATCHING3 R Extension command (IBM Corp., Armonk, NY, USA) and GraphPad Prism version 8.0 (GraphPad Software, Inc., La Jolla, Ca, USA) unless otherwise indicated.

## Results

### Respiratory mechanics of AE-ILD-UIP

Over the study period a total of 21 patients with AE-ILD-UIP underwent MV. Of these, 10 patients were analyzed according to inclusion criteria (see Additional file 1: Supplement). All of them died while on MV.

**Fig. 1:**
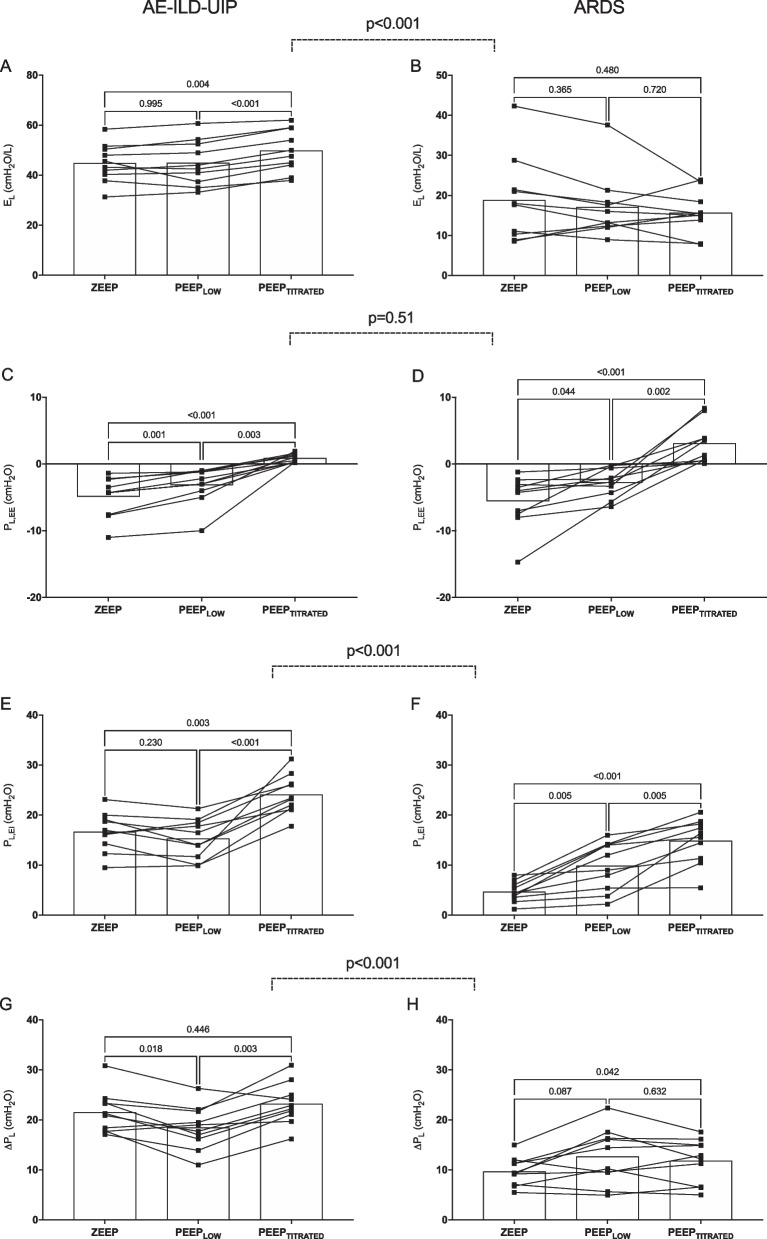
Measured individual values of *E*_L_, *P*_L,EE_, *P*_L,EI_ and Δ*P*_L_ the matched study groups at ZEEP, PEEP_LOW_ and PEEP_TITRATED_ phase. When testing as an interaction for whether the change in physiological variables at different PEEP levels was different between AE-ILD-UIP and ARDS (dotted *p*-values line), statistical difference was found for *E*_L_ (*p* < 0.001, panel **A** and **B**), *P*_L,EI_ (*p* < 0.001, panel **E** and **F**, *p* < 0.00) and Δ*P*_L_ (< 0.001, panel **G** and **H**). *E*_L_, lung elastance; *P*_L,EI_, end-inspiratory transpulmonary pressure; *P*_L,EE_, end-expiratory transpulmonary pressure; *P*_L_, transpulmonary driving pressure; ZEEP, zero positive end-expiratory pressure; PEEP, positive end-expiratory pressure; AE-ILD-UIP, acute exacerbation of interstitial lung disease with usual interstitial pneumonia pattern; ARDS, acute respiratory distress syndrome

Respiratory mechanics of AE-ILD-UIP at different PEEP levels are reported in Table 1, while changes in respiratory mechanics at different levels are shown in Fig. 1 (and Additional file 1: eFigure 2, Supplement). At ZEEP the median lung elastance (*E*_L_) was 44.4 cmH_2_O/L, transpulmonary driving pressure (Δ*P*_L_) was 21.1 cmH_2_O, *P*_L,EE_ was − 4.3 cmH_2_O and end-inspiratory transpulmonary pressure (*P*_L,EI_) was 16.7 cmH_2_O (Table 1). During the PEEP_LOW_ phase *P*_L,EE_ remained below 0 cmH_2_O (Table 1), median *E*_L_ and *P*_L,EI_ did not change (Fig. 1, panel A and E) while Δ*P*_L_ significantly decreased from baseline (*p* = 0.018,). During the PEEP_TITRATED_ phase *P*_L,EE_ was 0.8 cmH_2_O (Table 1) and *E*_L_ significantly increased as compared to both ZEEP and PEEP_LOW_ (*p* = 0.04 and *p* < 0.0001, respectively, Fig. 1, panel A), while *P*_L,EI_ and Δ*P*_L_ were higher as compared to PEEP_LOW_ (*p* < 0.001 and *p* = 0.003, respectively, Fig. 1, panel E and G).

**Table 1:** Blood gas analyses and partitioned respiratory mechanics of the AE-ILD-UIP and the ARDS population at different PEEP levels. Data are presented as median value and IQR

Variable	AE-ILD-UIP	ARDS	*p*-value
***ZEEP phase***			
*E*_L_, cmH_2_O/L	44.4 (39.7–50.7)	17.9 (9.9–23.3)	< 0.0001
*E*_cw_, cmH_2_O/L	3.2 (2.5–5.7)	5.4 (4–7.4)	0.12
*E*_tot_, cmH_2_O/L	49 (43.9–54.7)	22 (16.8–28)	< 0.0001
*P*_L,EI_, cmH_2_O	16.7 (14.8–19)	4.4 (2.9–6.3)	< 0.0001
*P*_L,EE_, cmH_2_O	− 4.3 (− 7.6– − 2.3)	− 4.1 (− 7.6– − 2.9)	0.66
Δ*P*_aw_, cmH_2_O	16.8 (13.8–19.3)	14.4 (11.5–21.2)	0.56
Δ*P*_L_, cmH_2_O	21.1 (17.8–23.6)	9.3 (7–11.5)	< 0.0001
pH, value	7.42 (7.41–7.42)	7.4 (7.37–7.41)	0.07
pO2, cmH_2_O	74 (66.5–80)	83 (75–89)	0.3
pCO2, cmH_2_O	39.5 (38–45)	39 (36.8–40)	0.06
***PEEP***_***LOW***_*** phase***			
*E*_L_, cmH_2_O/L	43.3 (36.8–53)	14.6 (12.2–19.1)	< 0.0001
*E*_cw_, cmH_2_O/L	3.4 (2.3–5.6)	5.7 (4.3–8.3)	0.09
*E*_tot_, cmH_2_O/L	48.5 (40–56.8)	22.1 (19.1–25.2)	< 0.0001
*P*_L,EI_, cmH_2_O	15.3 (11.3–18.7)	10.5 (5–14)	0.01
*P*_L,EE_, cmH_2_O	− 2.6 (− 4.3– − 1.2)	− 2.5 (− 4.6– − 0.5)	0.75
Δ*P*_aw_, cmH_2_O	16.8 (14.3–18.6)	15.1 (11.9–21.4)	0.9
Δ*P*_L_, cmH_2_O	18.4 (15.6–21.8)	12.3 (8.5–16.6)	0.02
PEEP, cmH_2_O	4 (4–4)	4 (4–5)	0.2
pH, value	7.41 (7.38–7.42)	7.4 (7.36–7.42)	0.3
pO_2_, cmH_2_O	93.5 (73.3–107)	74.5 (68.3–80.5)	0.1
pCO_2_, cmH_2_O	41 (38.5–42)	42 (39.8–47)	0.18
***PEEP***_***TITRATED***_*** phase***			
*E*_L_, cmH_2_O/L	48.8 (59–42.8)	15.2 (12.4–19.7)	< 0.0001
*E*_cw_, cmH_2_O/L	3.7 (3.2–5.9)	5.7 (4.7–7.2)	0.1
*E*_tot_, cmH_2_O/L	55.3 (45.9–62.5)	20.6 (19–24.5)	< 0.0001
*P*_L,EI_, cmH_2_O	23.3 (21.3–26.7)	16.9 (13.5–19.2)	0.001
*P*_L,EE_, cmH_2_O	0.8 (0.3–1.5)	2.4 (0.6–4.9)	0.04
Δ*P*_aw_, cmH_2_O	19.1 (16.1–21.6)	15.3 (9.4–17)	0.01
Δ*P*_L_, cmH_2_O	22.6 (20.8–25.8)	13.9 (6.6–16.5)	0.0001
PEEP, cmH_2_O	12 (10–14)	14 (12––17.5)	0.03
pH, value	7.38 (7.35–7.4)	7.37 (7.34–7.4)	0.34
pO2, cmH_2_O	80 (58–93)	105 (80–134)	0.01
pCO2, cmH_2_O	42 (39.5–43.3)	45 (40–48)	0.08

*AE-ILD-UIP, acute exacerbation of interstitial lung disease with usual interstitial pneumonia pattern; ARDS, acute respiratory distress syndrome; IQR, interquartile range;* Δ*P*_L_, transpulmonary driving pressure; *P*_L,EI_, end-inspiratory transpulmonary pressure; *P*_L,EE_, end-expiratory transpulmonary pressure; Δ*P*_aw_, driving pressure; *E*_tot_, respiratory system elastance; *E*_cw_, chest wall elastance; *E*_L_, lung elastance;* PEEP, positive end-expiratory pressure*

### AE-ILD-UIP as compared with historical, matched, ARDS controls

AE-ILD-UIP and matched ARDS groups were similar for SAPS II score (Additional file 1: eTable 1, Supplement). Lung infection was the cause for developing ARDS in all patients.

PEEP_TITRATED_, but not PEEP_LOW_ setting, resulted in higher PEEP in ARDS () compared with AE-ILD-UIP (14 VS 12 cmH_2_O, *p* < 0.001). At ZEEP, ARDS patients had lower *E*_L_ (17.9 cmH_2_O/L, *p* ≤ 0.0001), *P*_L,EI_ (4.4 cmH_2_O, *p* < 0.0001), and Δ*P*_L_ (9.3 cmH_2_O, *p* < 0.0001) compared with AE-ILD-UIP. During the PEEP_LOW_ and the PEEP_TITRATED_ phases, ARDS patients still had lower *E*_L_ (14.6 cmH_2_O/L, *p* < 0.0001 and 15.2 cmH_2_O/L, *p* < 0.0001 respectively), *P*_L,EI_ (10.5 cmH_2_O, *p* < 0.0001 and 16.9 cmH_2_O, *p* = 0.001 respectively), and Δ*P*_L_ (12.3 cmH_2_O, *p* = 0.02 and 13.9 cmH_2_O, *p* = 0.0001 respectively) as compared to AE-ILD-UIP.

Figure 1 shows that during the PEEP trial *E*_L_, *P*_L,EI_ and Δ*P*_L_ were different in AE-ILD-UIP and ARDS patients. *E*_L_ remained unchanged at PEEP_LOW_ and worsened at PEEP_TITRATED_ in AE-ILD-UIP, whereas it did not change in patients with ARDS (Fig. 1, panel A and B).

## Discussion

With this study, we report for the first time that AE-ILD-UIP patients under lung-protective MV strategy respond favorably in terms of respiratory mechanics to low PEEP levels, whereas respond unfavorably (and rather uniformly) to a *P*_es_-guided PEEP strategy to obtain positive *P*_L,EE_. The mechanical behavior of AE-ILD-UIP was different from that of matched “pulmonary” ARDS controls.

In our AE-ILD-UIP patients a low PEEP strategy resulted in reduction of Δ*P*_L_ indirectly suggesting alveolar recruitment, probably occurring in the areas of DAD superimposed to UIP. Indeed, despite we did not measure alveolar recruitment, we assume that in AE-ILD-UIP patients the lung regions spared by fibrosis but affected by DAD were likely de-recruited at ZEEP. Thus, it seems that the low PEEP strategy could be wise in patients with AE-UIP-ILD at least in terms of lung mechanics. These results are novel and referred to a cohort of patients rarely studied in the intensive care context. A previous study by Nava et al. assessed the respiratory mechanics during MV in seven patients with end-stage idiopathic pulmonary fibrosis [11] and reported values of lung elastance (46.1 cmH_2_O/L) similar to those found in our work. However, in that study lung mechanics were only measured at ZEEP.

Tailored PEEP titration in the context of lung-protective ventilation is still under debate [12]. During controlled MV, *P*_L,EE_ may be negative at ZEEP, indicating that the dependent lung regions are compressed [13]. This condition predisposes to tidal alveolar collapse and re-opening, resulting in high local shear forces that enhance VILI (atelectrauma) [14]. Negative *P*_L,EE_ is common in ARDS patients ventilated with lower PEEP levels in supine position and this largely explains the beneficial physiological effects of PEEP titration to achieve a positive *P*_L,EE_ reported in preclinical and clinical studies [15, 16]. Notwithstanding, the EPVent-2 trial showed that these positive physiological effects have to deal with the potential PEEP-induced lung injury caused by overdistension in the non-dependent lung regions [17]. We hypothesized the “Talmor” PEEP titration protocol in AE-ILD-UIP could lead to beneficial physiological effects also in patients with AE-ILD-UIP; however we found a rather sharp increase in *E*_L_ and Δ*P*_L_ in all of them (Fig. 1). It is tempting to speculate that *P*_es_-guided PEEP titration resulted in squishing among the patchy fibrotic tissue of the non-fibrotic lung regions (so called “*squishy ball lung”* phenomenon*)* [18] and that this effect invalidated the potential benefits of alveolar recruitment in the dependent lung regions.

Our study suffers from limitations: the small sample, the lack of quantitative analysis of hyper-inflated lung tissue [19] during PEEP titration and no end-expiratory lung volume assessment [20] allow only preliminary pathophysiological insights. Moreover, we did not assess the role of fluid balance as confounding factor. Finally, a selection bias should be acknowledged, as patients with AE-ILD are not usually placed on MV given the poor prognosis.

## Conclusions

In AE-ILD-UIP mechanically ventilated patients, low PEEP strategy may improve respiratory mechanics and, at difference with primary ARDS, PEEP titrated to obtain a positive *P*_L,EE_ significantly worsened lung mechanics. This paves the way to larger studies to clarify the best physiological response to PEEP in these patients. However, we feel that our findings could have practical implications when managing patients with AE-ILD-UIP under MV, suggesting that low PEEP strategy may be preferable to prevent lung injury.

### Supplementary Information

**Additional file 1**. Respiratory mechanics assessment protocol. Study algorithm and clinical and additional mechanical characteristics of the study population.

## Data Availability

Data are available at the Respiratory Disease Unit of the University Hospital of Modena, Italy, upon request.

## Abbreviations

ILD: Interstitial lung disease
AE-ILD: Acute exacerbation of ILD
UIP: Usual interstitial pneumonia
ARDS: Acute respiratory distress syndrome
AHRF: Acute hypoxic respiratory failure
bpm: Breaths per minute
MV: Invasive mechanical ventilation
ETI: Endotracheal intubation
NIV: Noninvasive mechanical ventilation
PEEP: Positive end-expiratory pressure
PBW: Predicted body weight
PSV: Pressure support
APACHE II: Acute physiology and chronic health evaluation II
SAPS II: Simplified Acute Physiology Score
IPF: Idiopathic pulmonary fibrosis
RICU: Respiratory intensive care unit
ICU: Intensive care unit
*P*_es_: Esophageal pressure
*P*_es,EI_: End-inspiratory esophageal pressure
*P*_es,EE_: End-expiratory esophageal pressure
*P*_L_: Transpulmonary pressure
Δ*P*_L_: Transpulmonary driving pressure
*P*_L,EI_: End-inspiratory transpulmonary pressure
*P*_L,EE_: End-expiratory transpulmonary pressure
*P*_plat_: End-inspiratory plateau pressure
Δ*P*_aw_: Driving pressure
*E*_tot_: Respiratory system elastance
*E*_cw_: Chest wall elastance
*E*_L_: Lung elastance
Vt: Tidal volume
IQR: Interquartile range
