# Supplementary material for: Physiological effects of lung-protective ventilation in patients with lung fibrosis and usual interstitial pneumonia pattern versus primary ARDS: a matched-control study
Source: Crit Care. 2023 Oct 18;27:398. doi: 10.1186/s13054-023-04682-5 (PMC10585808; doi:10.1186/s13054-023-04682-5)
Supplement: Supplementary file 1 — Additional file 1. Respiratory mechanics assessment protocol. Study algorithm and clinical and additional mechanical characteristics of the study population. [file 13054_2023_4682_MOESM1_ESM.docx]

**Supplementary materials**

According to our local protocol, sedation was achieved with a midazolam-propofol-remifentanil infusion to obtain a bispectral index between 40 and 60. Patients received cisatracurium to obtain myorelaxation. All subjects were placed in a 30° head up position.

In all patients, a multifunctional nasogastric tube equipped with an esophageal balloon was placed (NutriVent^TM^, nasogastric polyfunctional catheter; SIDAM, Mirandola, Italy), which was subsequently connected to a pressure transducer (OptiVent^TM^ monitor; SIDAM, Mirandola, Italy) to allow the assessment of P_es_ and the measurement of partitioned respiratory mechanics. The correct position of the esophageal catheter was confirmed by an end-expiratory occlusion test and with thoracic radiography^9^.

On admission, patients were ventilated with a Vt of 6 ml/kg of predicted body weight (PBW). The PBW of male patients was calculated as 50+0.91×(centimeters of height–152.4) and that of female patients as 45.5+0.91×(centimeters of height−152.4). PEEP was initially set between 5–8 cmH_2_O.

Respiratory mechanics were performed using the end-inspiratory occlusion technique during constant flow inflation and the end-expiratory occlusion method^10^.

All measurements of static respiratory mechanics were performed after 30 minutes of constant flow MV. The value of pressure was obtained during baseline ventilation with an airway occlusion at the end of expiration pressing the end-expiratory hold, until reaching a plateau pressure, and successively performing a similar procedure with an end-inspiratory occlusion. During the procedure, occlusions that did not produce a clean plateau were discarded (i.e., measures in which the airway pressure is not flat and has oscillations greater than 2-3 cmH_2_O).

Static respiratory mechanics variables were computed as follows:

1. End-inspiratory transpulmonary pressure = End-inspiratory plateau pressure – End-inspiratory esophageal pressure

P_L,EI_ = P_plat_ - P_es,EI_

1. End-expiratory transpulmonary pressure = Total end-expiratory airway pressure – End-expiratory esophageal pressure

P_L,EE_ = PEEP_tot_ - P_es,EE_

1. Driving pressure = End-inspiratory plateau pressure – total end-expiratory airway pressure

ΔP_aw_ = P_plat_ – PEEP_tot_

1. Tidal variation in esophageal pressure = End-inspiratory esophageal pressure – end-expiratory esophageal pressure

ΔP_es_ = P_es,EI_ – P_es,EE_

1. Transpulmonary driving pressure = Airway driving pressure – tidal variation in esophageal pressure

ΔP_L_ = ΔP_aw_ – ΔP_es_

1. Respiratory system elastance = Airway driving pressure/tidal volume

E_tot_ = ΔP_aw_/Vt

1. Chest wall elastance = Tidal variation in esophageal pressure/tidal volume

E_cw_ = ΔP_es_/Vt

1. Lung elastance = Transpulmonary driving pressure/tidal volume

E_L_ = ΔP_L_/Vt

**eFigure 1.**

*
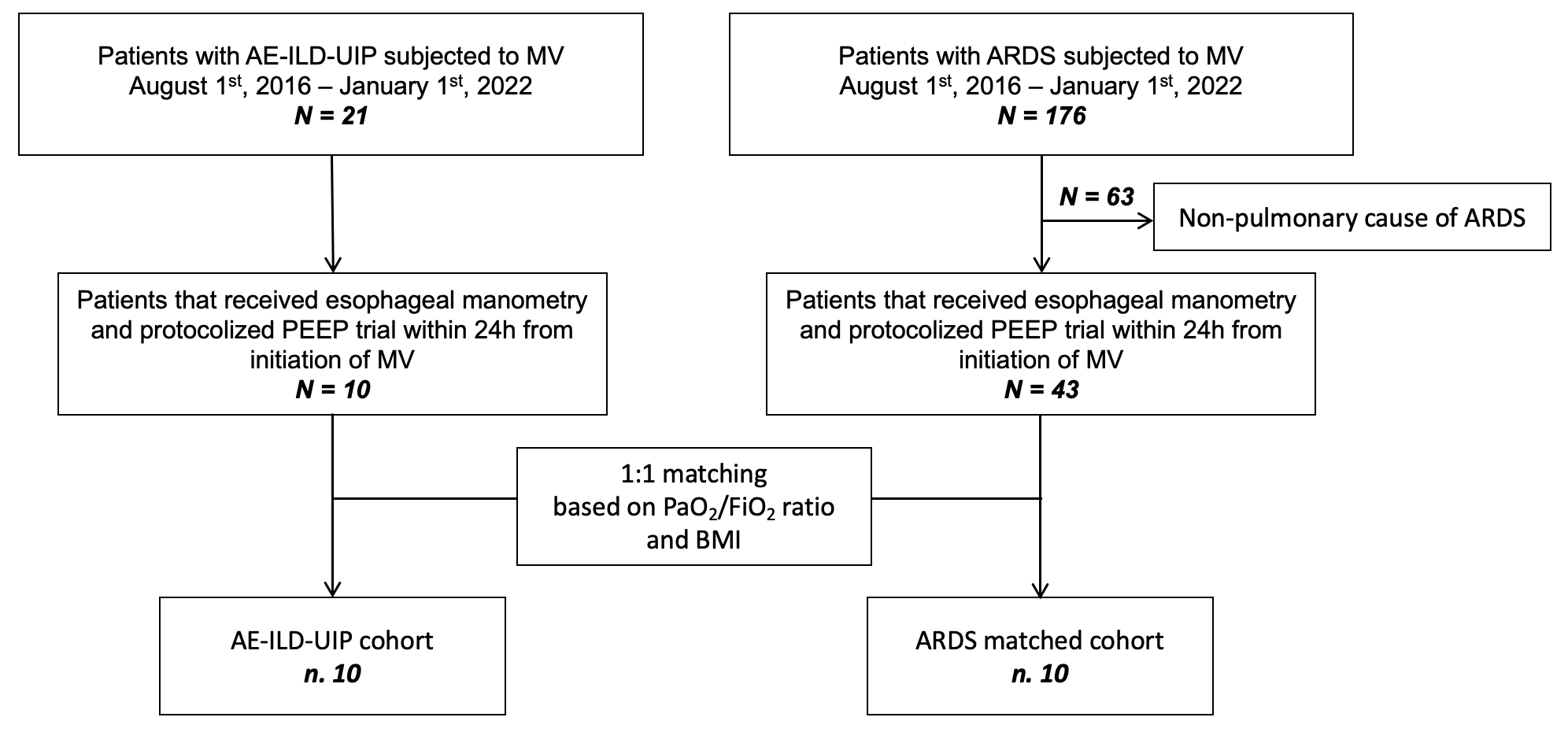
*

**eFigure 1.** Study algorithm.

*AE-ILD-UIP, acute exacerbation of interstitial lung disease with usual interstitial pneumonia pattern; ARDS, acute respiratory distress syndrome; RICU, Respiratory Intensive Care Unit; ICU, Intensive Care Unit; MV, mechanical ventilation; PEEP, positive end-expiratory pressure; P_L,EE_, transpulmonary end-expiratory pressure*

**eTable 1.**

| Parameter | All  (n=20) | AE-ILD-UIP  (n=10) | ARDS  (n=10) | p value |
| --- | --- | --- | --- | --- |
| **Age, years** | 68 (63.5 – 71.8) | 66.5 (63 – 68.9) | 69.5 (65 – 74) | 0.6 |
| **Male, n** | 15 (75) | 8 (80) | 7 (70) | 0.9 |
| **ILD diagnosis** |  |  |  |  |
| *IPF, n* | --- | 5 (50) | --- |  |
| *RA-ILD, n* | --- | 2 (20) | --- |  |
| *CHP, n* | --- | 2 (20) | --- |  |
| *U-ILD, n* | --- | 1 (10) | --- |  |
| *** PFTs** |  |  |  |  |
| *TLC, %pred* | --- | 71.5 (64 – 74) | --- |  |
| *FVC, %pred* | --- | 62.6 (58.5 – 71.2) | --- |  |
| *FEV1, %pred* | --- | 71 (60.5 – 77) | --- |  |
| *DLCO, %pred* | --- | 60 (48.3 – 68.8) | --- |  |
| **AE-ILD-UIP cause** |  |  |  |  |
| *Infectious* | --- | 6 (10) | --- |  |
| *Idiopathic* | --- | 4 (10) | --- |  |
| **Antifibrotic treatment, n** | --- | 9 (90) |  |  |
| **BMI, kg/m^2^** | 22.8 (21.3 – 24.2) | 23.7 (21.7 – 25) | 22.3 (20.8 – 23.4) | 0.7 |
| **Charlson index, score** | 3 (3 – 5) | 3 (3 – 5) | 4 (3 – 5) | 0.9 |
| **APACHE, score** | 13.5 (12.8 – 15) | 13.5 (12.3 – 14) | 13.5 (13 – 15) | 0.7 |
| **SAPS II, score** | 31.5 (27 – 36) | 31.5 (27 – 34.5) | 32 (27 – 37.5) | 0.5 |
| **†PaO_2_/FiO_2_, mmHg** | 96.5 (88.8 – 125.8) | 95.5 (90.5 – 122.5) | 98.5 (88.3 – 126) | 0.9 |
| **RICU/ICU survival, n** | 6 (70) | 0 (0) | 6 (60) | 0.02 |

**eTable 1.** General and clinical characteristics in the study groups on admission. Data are presented as number (n) and percentage for dichotomous values or median and IQR for continuous values and number and percentage.

* PFTs were available for 7 patients out of 10 with AE-ILD-UIP

† The values of APACHE score and PaO_2_/FiO_2_ ratio used for matching these groups were those measured at the time of RICU or ICU admission

*AE-ILD-UIP, acute exacerbation of interstitial lung disease with usual interstitial pneumonia pattern; ARDS, acute respiratory distress syndrome; BMI, body mass index; APACHE II, Acute Physiology and Chronic Health Evaluation II; SAPS II; Simplified Acute Physiology Score; RICU, respiratory intensive care unit; ICU, intensive care unit; IQR, interquartile range; IPF, idiopathic pulmonary fibrosis; CHP, chronic hypersensitivity pneumonitis; RA-ILD, rheumatoid arthritis associated interstitial lung disease; U-ILD, undetermined interstitial lung disease; TLC = total lung capacity; FVC = forced vital capacity; FEV1 = forced expiratory volume in 1 second; DLCO = lung diffusion test for carbon dioxide*

**eFigure 2.**

**eFigure 2.** Measured individual values of E_CW_, E_TOT_, P_AW_ in the matched study groups at ZEEP, PEEP_LOW_ and PEEP_TITRATED_ phase. When testing as an interaction for whether the change in physiological variables at incremental PEEP value was different between AE-ILD-UIP and ARDS (dotted p-values line),, statistical difference was found for E_TOT_ (**panel C and D**, p<0.001) while no difference were reported for E_CW_ (**panel A and B**, p=0.179) and P_AW_ (**panel E and F**, p=0.201).

*E_tot_, respiratory system elastance; E_cw_, chest wall elastance; P_aw_, driving pressure; ZEEP, zero positive end-expiratory pressure; PEEP, positive end-expiratory pressure; AE-ILD-UIP, acute exacerbation of interstitial lung disease with usual interstitial pneumonia pattern; ARDS, acute respiratory distress syndrome*
